# The Potential Role of the T2 Ribonucleases in TME-Based Cancer Therapy

**DOI:** 10.3390/biomedicines11082160

**Published:** 2023-08-01

**Authors:** Paola Campomenosi, Lorenzo Mortara, Barbara Bassani, Roberto Valli, Giovanni Porta, Antonino Bruno, Francesco Acquati

**Affiliations:** 1 Laboratory of Molecular Genetics, Department of Biotechnology and Life Sciences, University of Insubria, Via J.H. Dunant 3, 21100 Varese, Italy; paola.campomenosi@uninsubria.it; 2 Genomic Medicine Research Center, University of Insubria, Via J.H. Dunant 3, 21100 Varese, Italy; roberto.valli@uninsubria.it (R.V.); giovanni.porta@unisubria.it (G.P.); 3 Immunology and General Pathology Laboratory, Department of Biotechnology and Life Sciences, University of Insubria, Via Monte Generoso 71, 21100 Varese, Italy; lorenzo.mortara@uninsubria.it; 4 Laboratory of Innate Immunity, Unit of Molecular Pathology, Biochemistry, and Immunology, Istituto di Ricovero e Cura a Carattere Scientifico (IRCCS) MultiMedica, Via G. Fantoli 16/15, 20138 Milan, Italy; barbara.bassani@multimedica.it; 5 Department of Medicine and Surgery, University of Insubria, Via J.H. Dunant 3, 21100 Varese, Italy; 6 Human Genetics Laboratory, Department of Biotechnology and Life Sciences, University of Insubria, Via J.H. Dunant 3, 21100 Varese, Italy

**Keywords:** T2 ribonucleases, alarmins, tumor microenvironment, tumor immunology, immunotherapy, cancer therapy

## Abstract

In recent years, there has been a growing interest in developing innovative anticancer therapies targeting the tumor microenvironment (TME). The TME is a complex and dynamic milieu surrounding the tumor mass, consisting of various cellular and molecular components, including those from the host organism, endowed with the ability to significantly influence cancer development and progression. Processes such as angiogenesis, immune evasion, and metastasis are crucial targets in the search for novel anticancer drugs. Thus, identifying molecules with “multi-tasking” properties that can counteract cancer cell growth at multiple levels represents a relevant but still unmet clinical need. Extensive research over the past two decades has revealed a consistent anticancer activity for several members of the T2 ribonuclease family, found in evolutionarily distant species. Initially, it was believed that T2 ribonucleases mainly acted as anticancer agents in a cell-autonomous manner. However, further investigation uncovered a complex and independent mechanism of action that operates at a non-cell-autonomous level, affecting crucial processes in TME-induced tumor growth, such as angiogenesis, evasion of immune surveillance, and immune cell polarization. Here, we review and discuss the remarkable properties of ribonucleases from the T2 family in the context of “multilevel” oncosuppression acting on the TME.

## 1. Introduction

Cancer is a complex and heterogeneous disease characterized by the uncontrolled growth and proliferation of aberrant cells [1,2]. However, accumulating evidence has revealed a key role of the tumor microenvironment (TME) in cancer growth control [3,4,5]. The TME is a dynamic and intricate ecosystem consisting of cellular (stromal compartment, immune cells, endothelial cells), extracellular, and soluble components that collectively influence cancer initiation, progression, and response to therapy. Noteworthy, the mutual interactions between tumor cells and the TME components contribute to tumor growth, angiogenesis, immune evasion, and metastasis [3,4,5].

Cancer-associated fibroblasts (CAFs) represent one of the major cellular components of the TME. CAFs consist of activated fibroblasts that exhibit diverse phenotypes and functions. They contribute to tumor progression by secreting growth factors and cytokines, and remodeling the ECM, supporting tumor cell proliferation, invasion, and angiogenesis [6,7].

Another relevant host-related cellular component of the TME is represented by endothelial cells, the fundamental unit of angiogenesis. Angiogenesis, the formation of new blood vessels, is crucial for tumor growth and metastasis [8,9]. Endothelial cells within the TME secrete pro-angiogenic factors, allowing the recruitment and remodeling of blood vessels to sustain the nutrient supply and oxygenation required by rapidly growing tumors [8,9].

Infiltrating immune cells within the TME, including tumor-associated macrophages (TAMs), neutrophils, myeloid-derived suppressor cells (MDSCs), NK cells, and regulatory T cells (Tregs), profoundly influence tumor immune surveillance and anti-tumor immunity as well [10,11]. Based on immune cell plasticity and the subsequent response to diverse soluble factors present in the TME, the crosstalk between immune cells and tumor cells can result in either an immune-activating or immune-suppressive environment, influencing tumor progression and response to immunotherapy.

Finally, the TME is characterized by de-regulation of several molecular signaling pathways, such as the IL-6/STAT3 axis (as an example for the cytokine/growth factor axis) [12,13], TGFβ [14,15], and adenosine [16,17], together with profound environmental alterations, such as increased acidity [18,19], due to overproduction of lactate and subsequent metabolic adaptation, and extracellular matrix (ECM) remodeling [17,20], which further participate to tumor initiation and progression.

Based on these considerations, different TME-targeting approaches have been envisaged and applied in the last decades, which are here briefly summarized [21,22,23]:

(i) Reprogramming the immune landscape: immunotherapies, such as those relying on immune checkpoint inhibitors (ICIs), have emerged as a groundbreaking approach to modulating the TME. ICIs, including antibodies targeting programmed cell death protein 1 (PD-1), programmed death-ligand 1 (PD-L1), and cytotoxic T-lymphocyte-associated protein 4 (CTLA-4), unleash the immune system to recognize and attack cancer cells [24,25,26]. These therapies have shown remarkable success in treating several malignancies, highlighting the significance of the TME in immune evasion.

(ii) Stromal disruption: CAFs represent major stromal components in the TME that promote tumor growth and metastasis. Targeting CAFs can disrupt the TME’s supportive functions for cancer growth [27,28]. Strategies such as small-molecule inhibitors [29,30] and nanoparticle-based delivery systems [31,32] targeting CAF-specific markers have shown promising preclinical results. Additionally, fibroblast activation protein-alpha (FAP) inhibition has emerged as a potential therapeutic strategy to disrupt the stromal compartment [33,34,35].

(iii) Modulating angiogenesis: angiogenesis, the formation of new blood vessels, is essential for tumor growth and metastasis. Anti-angiogenic therapies, such as those based on vascular endothelial growth factor (VEGF) inhibitors, have been developed to inhibit tumor angiogenesis and normalize the abnormal vasculature in the TME [36,37]. Combining anti-angiogenic agents with other treatment modalities has improved outcomes in specific cancer types [38,39].

(iv) Targeting tumor metabolism: cancer cells exhibit altered metabolism, known as the Warburg effect, which influences the TME [40,41]. Targeting metabolic pathways in cancer cells and associated stromal cells holds promise as a therapeutic strategy. Inhibitors of glycolysis and glutamine metabolism enzymes, such as 2-deoxyglucose (2-DG) and glutaminase inhibitors, are being investigated for their potential to disrupt tumor metabolism and inhibit tumor growth [41].

Immunotherapy has become a revolutionary tool to fight cancer by exploiting the host defense, which is based on two concepts: (i) unlock/remove the brakes on the immune system, generated by the direct and indirect interaction with tumor cells and (ii) activate and direct the host immune system to better recognize and eliminate tumor cells. Several cell-based (both autologous and heterologous), antibody-based, molecular-based, and vaccine-based approaches have been developed for immunotherapy. Immunotherapy has generated some success in hematological malignancies and some solid cancers, such as melanoma and lung cancer.

Within this success, there is still a large window of unsuccessful immunotherapy for different tumor types. As another critical point, immunotherapy is not free from side effects, similar to chemotherapy and targeted therapies.

Given the current state of limited success in TME-based cancer therapy and the recently acknowledged limitations in the field of cancer immunotherapy [42], the identification and development of molecules acting at multiple levels within the TME (and possibly, at both cell-autonomous and non-cell-autonomous levels) would provide a potential avenue of great value for cancer treatment.

Within this frame, this review aims to provide a detailed survey of the experimental evidence gathered in the last decades to support a role for T2 RNases (and particularly human RNASET2) as multi-faceted, pleiotropically acting tumor suppressor proteins based on their role in establishing functional crosstalk between cancer cells and the TME.

## 2. Literature Search Strategy

A total of 124 articles were included in this review. They focus on the role of the TME in cancer development, the state-of-the-art in therapeutic approaches for TME-based cancer therapy, the general properties of several members of the T2 ribonuclease family, and, finally, the experimental evidence supporting a role for T2 RNases as potent, pleiotropically acting antitumoral agents. The bibliographic databases used to select the above-mentioned articles were PubMed and Google Scholar, covering more than six decades of experimental research (from 1957 to 2023). Our search strategy was based on using combinations of carefully selected subject headings (MeSHs) and keywords, such as “Tumor microenvironment”, “cancer therapy”, “immunotherapy for cancer”, “cancer angiogenesis”, “transferase-type ribonucleases”, “T2 Ribonucleases”, and “RNASET2”.

## 3. T2 RNases: General Features

The term RNaseT2 was originally assigned to an adenylic acid-preferential ribonuclease discovered in *Aspergillus oryzae,* a filamentous fungus commonly used in East Asia to saccharify rice, sweet potato, and barley [43]. RNaseT2 was later reported as a glycoprotein with a molecular mass of about 36 kDa, endowed with the ability to release 3′adenylic acid from yeast RNA at preferentially acidic pH [44]. Together with ribonucleases belonging to the A and T1 families, T2 enzymes represent transferase-type ribonucleases, which catalyze the cleavage of single-stranded RNA molecules through a 2′-3′ cyclic phosphate intermediate [45].

Despite their similarity in the catalytic mechanism, T2 ribonucleases display several features that clearly distinguish them from class A and T1 RNases. For instance, whereas T1 RNases are mainly restricted to bacteria and fungi and A RNases to metazoan, T2 ribonucleases have been reported throughout most taxa, ranging from viruses to mammals. Moreover, T2 RNAses typically display an optimal activity at a pH of 4–6, which is clearly different from the alkaline or weakly acidic range typically shown by T1 and A RNases [45].

T2 RNases display a complex pattern of subcellular localization. In addition to being secreted extracellularly [46], they are found in the cyto- and peri-plasmic space in bacteria [47], vacuoles in yeast cells [48], vacuoles or the surface of stigmatic papillae and the stylar-transmitting tract in several plant species [47,49,50,51], and ER/Golgi apparatus, lysosomes, mitochondria and processing-bodies (P-bodies) in mammalian cells, including humans [52,53,54]. It is presumed that T2 RNases carry out their catalytic activity within most of these subcellular structures.

From a chemical–physical point of view, most RNases belonging to the T2 family display a molecular mass in the 25–40 kDa range, which is higher compared to that reported for many A and T1 ribonucleases [46]. Although most T2 RNases consist of a single polypeptide chain, a few examples of homodimers have been reported [46]. In keeping with the frequent observation of T2 RNase localization in the extracellular environment due to their active secretion, most of these enzymes show a complex pattern of N-glycosylation at the canonical consensus Asn-X-Thr/Ser sequence [55,56]. As previously mentioned, most members of the T2 RNase family represent “acid” enzymes, with an optimal pH for catalytic activity ranging from 4.0 to 6.0 [46]. Although the thermodynamic stability of T2 RNases has not been thoroughly investigated, the few experimental data available suggest that these enzymes display high thermal stability, with some members tolerating short incubation at 90 °C. Concerning natural inhibitors of T2 RNases, several studies have shown that divalent cations such as Cu^2+^ and Zn^2+^ represent potent inhibitors of these enzymes [57]. Several mononucleotides act as strong T2 RNase inhibitors as well [46]. The substrate specificity of T2 RNases has been reported to be quite relaxed: these enzymes are largely base non-specific, although most members of the family were reported to be adenylic or guanylic acid-preferential [43].

Crystal structures have been described for several members of the T2 RNase family from bacteria, fungi, plants, and humans [57,58,59,60,61,62,63,64]. Noteworthy, although a wide range of biological roles has been attributed to different members of the T2 RNase family, the reported three-dimensional structures of these enzymes in bacteria, fungi, plants, and mammals turned out to be highly conserved, displaying a typical α + β core structure with a peculiar fold made by seven α-helices and eight β-strands [65]. Moreover, a key functional feature of T2 RNases was described in most members of this family, consisting of two highly conserved motifs (dubbed CAS I and CAS II), which represent the catalytic sites [65]. Within both motifs, a key role in catalysis is played by two highly conserved histidine residues, which determine a significant or complete loss of catalytic function when replaced by other amino acids [45].

## 4. T2 RNases: Biological Roles

Despite the high degree of evolutionary conservation shown by T2 RNases across taxa and the general similarity in most members of this enzyme family at the structural level, three decades of intensive research have unveiled an impressive range of biological roles played by these proteins [66]. Noteworthy, in many instances, the biological functions ascribed to these enzymes turn out to be independent of their ribonuclease activity [65].

**Figure 1:**
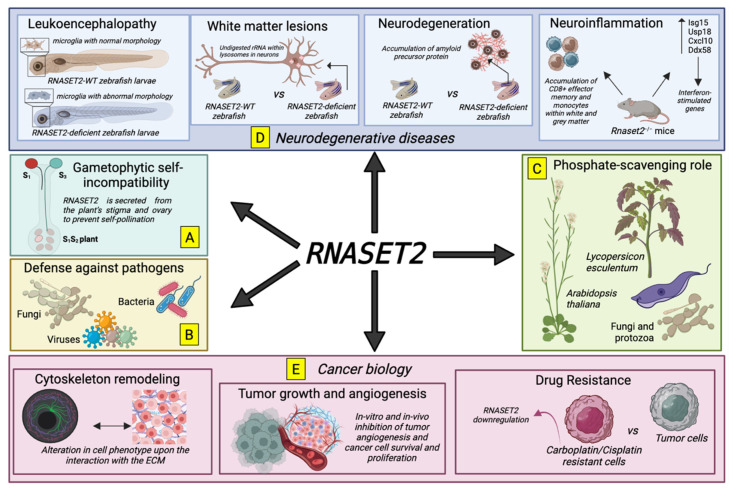
Pleiotropic activities of RNASET2 in pathophysiology. (**A**) RNASET2 prevents the fertilization of the same plant. (**B**) RNASET2-mediated response against infections induced by bacteria, viruses, and fungi. (**C**) Phosphate-scavenging activities of RNASET2 found in plants (*Arabidopsis thaliana* and *Lycopersicon esculentum*), fungi, and protozoa. (**D**) RNASET2 contribution to neurodegenerative diseases, where RNASET2 deletion drives abnormal microglia, alteration in waste rRNA digestion, accumulation of amyloid precursors, and overall neuroinflammation. (**E**) RNASET2 role in cancer in tumor cell cytoskeleton remodeling and subsequent interactions with ECM, tumor growth and angiogenesis, and drug resistance against chemotherapy (Carboplatin and Cisplatin). The figure was prepared using Biorender illustration software (https://www.biorender.com/, accessed on 17 July 2023).

Among the many roles reported for T2 RNases, the regulation of gametophytic self-incompatibility (GSI) by S-RNases in several plant species was described first. S-RNases represent the pistil component responsible for GSI in plants belonging to the Rosacee, Solanacee, and Plantaginaceae taxonomic groups [66]. GSI is a complex process preventing self-fertilization in these plants, and it was shown to be dependent on the highly polymorphic *S*-locus encoding a T2 RNase. These enzymes are secreted from the plant’s stigma and ovary into the extracellular environment where pollen tubes grow, and if the genotype of both pollen and pistil match, they enter the pollen tube to degrade ribosomal rRNA (rRNA) and prevent self-pollination (Figure 1A) [67].

The observed cytotoxic activity of S-RNases prompted several research groups to evaluate the hypothesis that GSI might represent an evolutionary innovation implemented in some plants by exploiting an ancient ribonuclease previously involved in host defense against pathogens [66]. In keeping with this model, a bulk of experimental evidence has now been gathered to support such a role for T2 RNases. Indeed, these enzymes were later found to be strongly induced in response to bacterial and viral infections in a wide range of organisms, from plants to fungi and invertebrates (Figure 1B) [68,69,70,71]. Moreover, a direct antimicrobial or antiparasite activity has been reported for some members of the T2 RNase family (Figure 1B) [72,73].

A completely unrelated role for T2 RNases was later reported, which involved the scavenging of extracellular RNAs in starving conditions. Indeed, T2 RNases were found to be induced following phosphate starvation in several plant species, possibly to provide phosphates from RNA molecules [74,75,76]. Of note, a putative phosphate-scavenging role for T2 RNases was suggested in fungal species and protozoa as well (Figure 1C) [77,78]. Possibly related to the involvement of T2 RNases in phosphate scavenging is their putative housekeeping role in nucleotide metabolism. In this context, in addition to the up-regulation of the *Arabidopsis thaliana* T2 RNAse *RNS2* under phosphate starvation, the same gene was shown to be expressed constitutively in all tissues, and *RNS2* mutants displayed intracellular RNA accumulation in the vacuole coupled to constitutive ribophagy [79], leading to the suggestion of a housekeeping role for *RNS2* in ribosomal turnover (Figure 1C). In keeping with this hypothesis, RNASET2 zebrafish was found to be localized in lysosomes, and mutants lacking enzyme activity displayed enlarged lysosomes accumulating rRNA in brain cells [80].

The role of RNASET2 has been exhaustively demonstrated in the context of neurodegenerative diseases; a selection of these activities is illustrated in Figure 1D.

Human infantile-onset *RNASET2*-deficient cystic leukoencephalopathy is a Mendelian disorder that mimics cytomegalovirus brain infection during fetal development, resulting in inflammatory brain lesions (Figure 1D). Weber et al. utilized a zebrafish model deficient in *RNASET2* to investigate the effects of a lack of this enzyme [81]. The authors observed that *RNASET2*-deficient zebrafish larvae exhibited an increased number of microglia with abnormal morphology, often containing neuronal inclusions (Figure 1D) [81]. Furthermore, they found enhanced lysosomal staining within specific populations of the myeloid cell lineage, including microglia. This study offers valuable insights into the pathogenetic mechanisms underlying the prenatal onset of *RNASET2*-deficient leukoencephalopathy in humans, establishing a connection between this lysosomal disorder and the innate immune system (Figure 1D) [81]. Additionally, it highlights the potential links between RNASET2 deficiency and other immune-related childhood encephalopathies such as Aicardi-Goutières syndrome (AGS).

Another study by Hamilton et al., using a mutant zebrafish lacking Rnaset2, showed an accumulation of undigested rRNA specifically within lysosomes in neurons of the brain. Using high-field intensity magnetic resonance microimaging, the authors identified white matter lesions in the zebrafish, able to recapitulate the same lesions observed in infants with RNASET2 deficiency (Figure 1D) [82]. Notably, the authors found a correlation between the accumulation of amyloid precursor protein and astrocytes at sites of neurodegeneration (Figure 1D) [82].

Moving to murine models, Kettwig M. et al. generated *Rnaset2*^−/−^ mice using the application of CRISPR/Cas9-mediated genome editing [83]. The *Rnaset2*^−/−^ mice exhibited an upregulation of interferon-stimulated genes, accompanied by IFNAR1-dependent neuroinflammation. This neuroinflammation was characterized by the infiltration of CD8^+^ effector memory T cells and inflammatory monocytes into both the grey and white matter of the brain (Figure 1D) [83]. By utilizing single-nucleus RNA sequencing, the authors identified dysfunctions in glial cells and neurons that disrupted brain homeostasis (Figure 1D) [83].

A case report performed using brain imaging analysis on a 5-month-old patient with motor delay, neurological regression, infrequent seizures, and microcephaly revealed the presence of white matter abnormalities, calcification, and cysts in the anterior temporal region [84]. To investigate the underlying cause, the authors performed a screening for mutations in the *RNASET2* and *RMND1* genes together with a comprehensive review of previously reported cases of individuals with RNASET2-deficient leukodystrophy, comparing their clinical data and magnetic resonance imaging (MRI) with the observation reported in the case report. Using this analysis, the authors identified a novel homozygous variant of c.233C > A; p.Ser78Ter in exon 4 of the RNASET2 gene compatible with the diagnosis of RNASET2-deficient leukoencephalopathy [84].

Further characterization of the human *RNASET2* gene unveiled novel roles for this class of enzymes, such as the inhibition of cell growth in vitro and in vivo, the induction of apoptosis, a marked remodeling of the cytoskeleton, the inhibition of angiogenesis and fine regulation of the immune response, as will be reported in the following sections. Of note, most of these roles are closely linked to cancer development (Figure 1E).

The *RNASET2* gene represents the only human member of the highly conserved family of T2/Rh/S extracellular ribonucleases [45]. Noteworthy, as already mentioned, T2 ribonucleases display an impressive degree of evolutionary conservation, suggesting a crucial and very ancient biological role for this class of enzymes [65]. Furthermore, a great bulk of experimental evidence has been gathered in the last decades to suggest a marked oncosuppressive activity of the human RNASET2 protein in both in vivo and in vitro experimental settings (Figure 1E) [85]. Noteworthy, negative control of cell growth, induction of apoptosis, and a marked remodeling of the cell cytoskeleton (all representing biological processes deeply involved in tumor suppression) were consistently reported to be modulated by several members of the T2 RNase family from evolutionarily distant species, ranging from *Saccharomyces cerevisiae* to *Homo sapiens.*

Even more relevant are recent data strongly supporting a significant (although not exclusive) contribution of the immune system in RNASET2-mediated tumor suppression, due to the ability of this enzyme to finely tune key immune processes and trigger a powerful anticancer response, likely by acting as an alarmin-like molecule [86]. Although this immune-based response was shown to be mainly based on the ability of RNASET2 to modulate the recruitment and polarization pattern of macrophages, there is preliminary evidence pointing at other cellular effectors of both the innate and adaptive immune system being likely involved in a complex RNASET2-based interplay between cancer cells and the immune system [87].

## 5. T2 RNases as Evolutionarily Conserved Tumor Suppressors

The human *RNASET2* gene (previously known as *RNASE6PL*) was identified in 1997 [88], in the frame of a positional cloning strategy aimed at identifying putative tumor suppressor genes from chromosome 6q27, a region long known to undergo frequent rearrangements such as deletions and translocations in a wide range of human cancers [89,90,91,92,93,94].

The first experimental evidence pointing at a role for RNASET2 in tumor suppression came from a study on human ovarian carcinoma, which showed a marked downregulation of this gene’s expression in a panel of ovarian cancer cell lines and primary tumors, coupled with the ability of RNASET2 to inhibit tumor growth in vivo when overexpressed in several cancer cell lines following their inoculation in xenograft mouse models (Figure 1E) [95]. Strikingly, a mutational screening in the panel of ovarian cancers above described did not show the occurrence of mutations predicted to significantly affect the RNASET2 protein coding region. Rather, frequent occurrence of *RNASET2* down-regulation at the transcript level was reported in the same panel of cancer samples.

**Figure 2:**
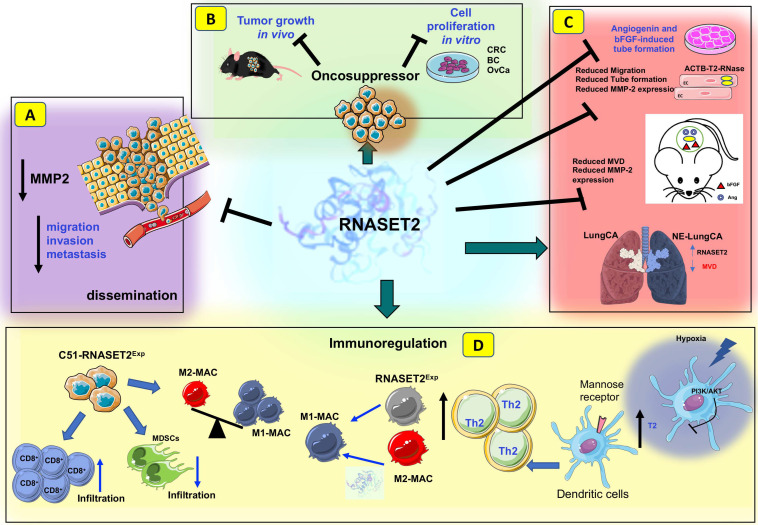
Multiple activities of RNASET2 in the tumor microenvironment. (**A**) RNASET2-overexpressing tumor cells are characterized by decreased proliferation capabilities, as observed in colorectal (CRC), breast (BC), and ovarian cancer (OvCa) cell lines. The same behavior was observed in RNASET2-overexpressing tumor cells injected into mice. (**B**) RNASET2-overexpressing tumor cells exhibit reduced migration and invasion capabilities, associated with impaired metastatization, when transferred into mice. RNASET2 has also been associated with anti-tumor activities by acting on the tumor microenvironment at multiple levels. (**C**) The administration of RNASET2 recombinant protein to human umbilical-vein endothelial cells (HUVECs) limits their capability to generate capillary-like structures in vitro. Also, mice receiving gels/sponges supplemented with bFGF or angiogenin, when associated with RNASET2 protein, show a reduction in their microvascular density. Finally, neuroendocrine lung neoplasms, characterized by increased expression of RNASET2, have reduced microvascular density compared to other lung tumor types. (**D**) Immunoregulatory activities of the secreted RNASET2 molecule. Increased expression of RNASET2 in monocyte-derived dendritic (mo-DCs) cells after hypoxia results in increased DC activation. Also, RNASET2 has been reported to prime host dendritic cells (DCs) to induce an infection permissive Th2 response both in vitro and in vivo, via mannose receptor activation. RNASET2 knockdown in THP-1 differentiated macrophages supports the induction of pro-inflammatory M1 macrophages; when M2 macrophages are exposed to RNASET2, as a recombinant protein, they undergo M1 polarization. The injection of RNASET2-overexpressing C51 colon cancer cell line into immunocompetent mice results in the expansion of M1 macrophages, together with increased infiltration of CD8^+^ T cells, while reducing the accumulation of MDSCs. The figure was prepared using part of the graphical art available in Servier medical art (https://smart.servier.com/, accessed on 20 May 2023), following modifications required to generate the cartoon.

Subsequent investigations confirmed the tumor-suppressive role of RNASET2 in ovarian cancer models by showing its ability to suppress the metastatic potential of a highly invasive and aggressive cancer cell line in vivo [96]. Strikingly, both tumor and metastasis suppression were shown to be unaffected following overexpression of a catalytically dead mutant of *RNASET2,* suggesting that both phenomena apparently represent ribonuclease-independent processes [96]. The putative role of RNASET2 in human ovarian cancer suppression was later supported by a report showing a marked downregulation of RNASET2 expression in both carboplatin- and cisplatin-resistant A2780 ovarian cancer cell lines compared to their parental counterparts (Figure 1E) [97]. Consistent with this finding, increased *RNASET2* expression at the transcript level was associated with longer overall survival in epithelial ovarian cancer patients [98]. Furthermore, an extension of the above-mentioned studies to other cellular models of human ovarian cancer confirmed the ability of RNASET2 not only to negatively affect cell proliferation and clonogenic activity but also to confer a cell phenotype strictly dependent on the modulation of the tumor cell–extracellular matrix interaction, which, in turn, resulted in decreased oncogenic Src signaling (Figure 1E) [98]. Although most of the above-mentioned studies mainly focused on cancer-related changes in *RNASET2* expression assessed at the RNA level, more recently, a post-transcriptional mechanism for RNASET2 downregulation in cancer cells was reported as well. The F-box FBOX6 protein (a subunit of the SCF complex) was found to act as a ubiquitin E3 ligase responsible for proteasome-mediated RNASET2 degradation in ovarian cancer models in vitro. A direct FBOX6–RNASET2 interaction was demonstrated to be essential for FBOX6-mediated RNASET2 degradation, and FBOX deficiency was found to stabilize RNASET2 protein levels, leading to suppression of ovarian cancer cell proliferation, migration, and invasion (Figure 2A,B). In keeping with these findings, FBOX6 was found to be overexpressed in ovarian cancer, where it is associated with poor survival rates [99].

To evaluate whether the long-established evolutionary conservation of T2 ribonucleases might also act at the functional level, the effect of the fungal T2 RNase ACTIBIND from *Aspergillus niger* on cancer growth was also investigated. ACTIBIND was shown to inhibit the clonogenic potential of several human cell lines derived from colon, breast, and ovarian carcinomas independently of its catalytic activity, possibly through its ability to affect their morphology and motility (Figure 2C) [100]. Furthermore, xenograft assays carried out with the human colon cancer-derived HT-29 cell line showed that recombinant ACTIBIND, administered either as a subcutaneous or intraperitoneal treatment, displayed a marked oncosuppressive activity in vivo. Of note, by carrying out both preventive and therapeutic in vivo tumor growth assays, orally administered ACTIBIND was also found to drastically reduce the growth of colorectal cancers in carcinogen-treated rats (Figure 2C) [100]. Strikingly, when these assays were carried out by replacing ACTIBIND with human RNASET2, the oncosuppressive activity of the latter was consistently confirmed (Figure 2C) [100].

The striking evolutionary conservation of T2 RNases at the functional level was further validated with investigations of the yeast Rny1p T2 RNase. In this model system, oxidative stress was shown to trigger the release of Rny1p from the vacuole into the cytoplasm, followed by cell death. Once again, modulation of yeast cell survival by Rny1p turned out to be independent of its catalytic activity. Noteworthy, the phenotype of increased viability under oxidative stress observed in a yeast Rny1p knock-out strain was rescued by human *RNASET2* overexpression, clearly suggesting an impressive degree of functional conservation for this class of proteins over extremely long evolutionary timeframes [48].

Later, an unprecedented oncosuppressive role was indirectly suggested by the finding of a strong inhibition of human *RNASET2* expression by the Tax oncoprotein from the HTLV-1 retrovirus, which is responsible for human adult T-cell leukemia [101].

In keeping with the data obtained with yeast’s Rny1p T2 RNase, independent lines of research reported new data further supporting the role of T2 RNases as stress response genes, a feature that is commonly found in tumor suppressor genes. For instance, Wang et al. showed that different kinds of stress conditions led to human RNASET2 overexpression followed by an increased apoptotic rate in both primary keratinocytes and melanocytes [102]. Noteworthy, stress-induced *RNASET2* upregulation in both cell types was found to trigger apoptosis through a direct interaction between RNASET2 and tumor necrosis factor receptor-associated factor 2 (TRAF-2), a key intermediate modulator of tumor necrosis factor α (TNF-α).

A wider range of cellular stresses was subsequently found to trigger a marked increase in RNASET2 expression and secretion in several ovarian cancer cell lines, and stress-induced RNASET2 overexpression was in turn associated with decreased clonogenic potential and decreased growth in soft agar [103]. The migration and adhesion patterns were also found to be strongly affected by RNASET2 expression levels in stress-exposed OVCAR3 human ovarian cancer cells [103].

Furthermore, RNASET2 was reported to be involved in oxidative stress-mediated cell death in CHO cells, by acting as a mediator of Reactive Oxygen Species (ROS) generation during lipotoxicity [104].

Recently, an unexpected finding suggested a novel mechanism of oncosuppression for human *RNASET2*, occurring in the mitochondria. Liu et al. reported for the first time a role for RNASET2 in processing or degrading mitochondrial RNAs within the intermembrane space [52]. Later on, the same research group found that the RNA component of mammalian telomerase (*TERC*) is imported into the mitochondria to be processed by RNASET2 and released as a shorter transcript (*TERC-53*), which is later exported back into the cytosol [105]. This suggested that cytosolic *TERC-53* might play a downstream signaling role in mitochondrial function. Noteworthy, experimental overexpression of cytosolic *TERC-53* was found to significantly increase the senescence rate in primary human embryonic lung fibroblasts, suggesting that a further, well-established antioncogenic process (cellular senescence) might be triggered by mitochondrial RNASET2-mediated production of *TERC-53* [106].

Taken together, the experimental data gathered in the last two decades strongly support the notion of T2 ribonucleases (and particularly human RNASET2) acting as highly pleiotropic tumor suppressor proteins, endowed with the ability to hamper tumor growth in a broad spectrum of human cancer types by many independent mechanisms.

## 6. T2 RNases as Modulators of Angiogenesis and Immune Responses

T2 ribonucleases have been described as extracellular enzymes that are actively secreted by cells expressing them [65]. In turn, this finding prompted further investigations focusing on the potential roles of T2 RNases outside cells. Given the long-established role of T2 RNases as oncosuppressors, the attention of several research groups turned to the potential role of this enzyme class in the regulation of cancer growth by means of modulating key cancer-related biological processes taking place in the TME.

The first evidence of a cancer-related process regulated by extracellular T2 RNases was reported by Roiz et al. [100], who found that treatment of human umbilical vein endothelial cells (HUVEC) with the recombinant T2 RNase ACTIBIND was associated with a marked decrease in both angiogenin- and bFGF-dependent angiogenesis, as assessed using in vitro tube formation assays (Figure 2C). Moreover, extracellular ACTIBIND was found to significantly affect the intracellular actin network in both HT-29 and ZR-75-1 human cancer cell lines. This actin-remodeling process was associated with a marked inhibition of HT-29 and ZR-75-1 invasion on Matrigel-coated filters. As previously reported for several cell-autonomous oncosuppressive roles ascribed to these enzymes, suppression of angiogenesis by extracellular T2 RNases was found to represent an evolutionarily conserved process as well, since recombinant human RNASET2 was shown to display a marked antiangiogenic effect on the same in vitro assays previously used for ACTIBIND (Figure 2C) [107].

Further studies strongly confirmed the role of human RNASET2 as an effective antiangiogenic factor since human recombinant RNASET2 was found to completely inhibit angiogenin-induced tube formation in HUVEC cells [108].

The antiangiogenic effect of T2 RNases was also demonstrated in vivo. Implantation of bFGF- or angiogenin-loaded Gelfoam sponges in nude mice showed significant stimulation of in vivo neoangiogenesis, which was markedly suppressed by either the addition of recombinant ACTIBIND to the sponges or the intraperitoneal injection of ACTIBIND. These results suggested that inhibition of angiogenesis might represent a potential mechanism for ACTIBIND-mediated in vivo tumor suppression (Figure 2C) [109].

In addition to angiogenesis, modulation of the immune system is another key process exploited by cancer cells to thrive within a hostile environment.

In 2009, two independent studies showed the ability of omega-1, the T2 RNase produced in the eggs of the helminth parasite *Schistosoma mansoni*, to prime host dendritic cells (DCs) and induce an infection-permissive Th2 response both in vitro and in vivo [110,111]. In later studies, extracellular omega-1 was found to bind and be internalized by DCs through the mannose receptor to impair protein synthesis and degrade specific mRNAs [112].

Further experimental evidence supporting a role for T2 RNases in DC biology was also reported in humans, where hypoxic stress was found to significantly increase RNASET2 expression and secretion by monocyte-derived DCs (Figure 2D) [113]. The increased secretion of RNASET2 by DCs under a typical cancer-related stress condition such as hypoxia was compatible with a role for this protein as an “alarmin-like” molecule, endowed with the ability to signal the innate immune system about potential danger and activate a defense response program. Noteworthy, RNASET2 induction by hypoxic stress in DCs was found to be inhibited by PI3K/AKT activation, suggesting a plausible link between activation of a pro-oncogenic signaling pathway and the concomitant inhibition of the tumor-suppressor role of RNASET2 (Figure 2D) [113].

In addition to dendritic cells, monocytes and macrophages were also defined as another key cellular component of the innate immune system regulated by T2 RNases (Figure 2D). Indeed, when high endogenous expression levels of human *RNASET2* were knocked-down with RNA interference in the human promyelocytic THP-1 cell line and the polarization pattern in THP-1 derived macrophages was investigated by assessing the expression pattern of several polarization markers, the silencing of *RNASET2* was found to be associated with a dramatic M1 to M2 polarization shift, suggesting a key role for RNASET2 in modulating the phenotype of mature macrophages (Figure 2D) [114]. Moreover, treatment of *RNASET2*-silenced macrophages with recombinant RNASET2 was able to partially rescue the observed polarization shift, suggesting that, in addition to *endogenous* RNASET2, the extracellular enzyme (possibly secreted by cancer cells under stress conditions) might regulate this process as well. Of note, unlike M2 cells, M1-polarized macrophages are known to play a powerful antitumoral role in both in vitro and in vivo experimental settings (Figure 2D) [115].

Further experimental evidence pointing at RNASET2 as a key activator of the innate immune system was reported by Gruelich et al., who demonstrated that lysosomal RNASET2 plays a key non-redundant role in producing RNA degradation products that act as ligands for Toll-like receptor 8 (TLR8) activation in human monocytes [116]. Taken together, these findings provide evidence supporting a role for RNASET2 as an alarmin-like molecule, endowed with the ability to provide an early and strong host defense response involving innate immunity. A later study confirmed the role of human RNASET2 in the regulation of the nucleic acid-sensing subclass of Toll-like receptors within macrophage endosomes/lysosomes. In that study, RNASET2 was reported to positively regulate the single-strand RNA-sensing TLR7 while downregulating double-strand RNA-sensing TLR3 [116].

Further evidence pointing at a conserved role for T2 RNases in modulating the innate immune system was reported in a study showing that human recombinant RNASET2 can induce a robust inflammatory response when injected into the body wall of the medicinal leech *Hirudo medicinalis*. Indeed, RNASET2-treated leeches showed marked infiltration of host cells expressing molecular markers specific for macrophages [68]. Furthermore, the injection of human RNASET2-soaked Matrigel pellets into the leech body wall led to massive recruitment of CD68^+^/AIF-1^+^ macrophages. Of note, when the leeches were challenged with either bacterial lipopolysaccharides (LPSs) or live cells from the pathogen *Micrococcus nishinomiyaensis*, massive recruitment of host leech macrophages displaying a marked increase in *endogenous* RNASET2 was observed. This suggested that, in addition to macrophage recruitment, RNASET2 might be involved in other cellular processes regulating macrophage biology. Moreover, leech granulocytes, another key effector cell type involved in innate immunity, were found to increase their endogenous RNASET2 expression levels following LPS injection [72]. Immunogold analysis of RNASET2 in LPS-treated leech granulocytes using transmission electron microscopy (TEM) revealed the presence of endogenous RNASET2 within the granules of these cells, suggesting that this RNase is a component of the armory of proteins secreted by LPS-activated granulocytes.

In recent years, independent evidence suggesting a role for T2 RNases in immune response regulation has come from the results of several genome-wide association studies (GWASs) focused on a wide range of human autoimmune diseases, which showed that several SNPs located in the 5′ region of the human *RNASET2* gene are highly associated with an increased risk of vitiligo, Graves’ disease, rheumatoid arthritis, Crohn’s disease and type I diabetes [86]. Strikingly, *RNASET2* expression was significantly upregulated in patients with several autoimmune diseases compared to healthy controls [86,117].

Taken together, data from the literature provide compelling evidence about the role of extracellular T2 RNases in the modulation of two key cancer-related processes taking place in the TME, namely angiogenesis and immune system modulation.

## 7. RNASET2-Mediated Tumor Suppression by TME Modulation In Vivo

Based on the data reported above, further studies were carried out to investigate the potential of T2 ribonucleases to suppress cancer growth in vivo by means of TME modulation.

The powerful in vivo oncosuppressive activity of ACTIBIND T2 RNase was reported in a xenograft-based cancer model, in which nude mice previously injected with the highly tumorigenic and metastatic human malignant melanoma A375SM cell line were treated with recombinant ACTIBIND. In this model, ACTIBIND-treated animals showed a marked delay in both tumor growth and metastasis compared to PBS-treated controls [109].

In keeping with these data, tumor-associated neovascularization (evaluated using a microvessel density assessment) was significantly reduced in suppressed tumors from ACTIBIND-treated mice compared to control animals, and the antiangiogenic effect of ACTIBIND was coupled to a marked increase in apoptosis and a significant reduction in MMP-2 expression within the tumor mass. Since ACTIBIND, like angiogenin, was found to be internalized in vitro by HUVEC cells, this finding prompted the authors to speculate a direct inhibition of angiogenin activity by ACTIBIND through a competitive mechanism. Indeed, ACTIBIND was found to inhibit both angiogenin-driven MMP-2 expression and rRNA transcriptional activity in HUVEC cells (Figure 2C) [109].

As already reported with in vitro assays, the antiangiogenic activity of ACTIBIND was later shown to represent a highly conserved functional feature of T2 RNases using in vivo assays as well. Human recombinant RNASET2 treatment of nude mice injected with HT29 or A3775SM human cancer cells was associated with a three-fold decrease in tumor volume compared to Avastin-treated animals, coupled with a significant decrease in mean blood vessel counts and higher survival rates [108].

In vivo studies published in the last decade clearly suggested a role for T2 RNases in immune response-mediated tumor suppression as well. For instance, ectopic expression of human *RNASET2* in the highly malignant *RNASET2*-nonexpressing HEY3MET2 ovarian cancer cell line was found to significantly suppress its tumorigenic potential in nude mice, independently from the protein’s catalytic activity [118]. Of note, a detailed histological analysis of tumor specimens unveiled a marked infiltration of host leukocytes in RNASET2-suppressed tumors, which proved to be mainly represented by cells belonging to the monocyte/macrophage lineage. The same outcome was observed by injecting HEY3MET2 cells ectopically expressing RNASET2 into another immunodeficient model, the Rag2/γc double knockout mice. Strikingly, when these mice were pre-treated with the in vivo macrophage-depleting agent clodronate, the tumorigenic potential of HEY3MET2 cells was largely rescued even in the presence of high RNASET2 levels, strongly suggesting an active role for host macrophages in RNASET2-mediated tumor suppression [118]. Furthermore, evaluation of the macrophage polarization pattern in untreated animals clearly showed that RNASET2-recruited host leukocytes belonged to the M1 subclass of macrophages, with potent antitumor activity [119,120].

Since protein overexpression was reported to potentially lead to unphysiological effects in transfected cells, the same research group later developed an independent and complementary xenograft-based model of ovarian cancer, in which the high *RNASET2*-expressing OVCAR3 ovarian cancer cell line was genetically engineered to completely knock-down endogenous *RNASET2* expression with RNA interference [121]. In keeping with the results from the previous in vivo model, RNASET2-silenced OVCAR3 cells showed a significant increase in their tumorigenic potential compared to their wild-type counterpart when challenged in nude mice, thus confirming the role played by human RNASET2 in in vivo tumor suppression. Moreover, a histological survey of tumor sections confirmed that RNASET2-suppressed tumors from control OVCAR3 cells were highly infiltrated with M1-polarized host macrophages, again pointing at RNASET2 as a potent in vivo modulator of the polarization pattern in this cell type. This conclusion was further supported by transcriptomic analysis of these tumors, showing that human genes within the tumor mass, whose expression was mainly affected following RNASET2 silencing in OVCAR3 cells, were enriched in GO/KEGG categories related to leukocyte activation, immunity, cell adhesion, and cancer. In contrast, the murine GO/KEGG categories found to be significantly affected by RNASET2 within the tumor transcriptome belonged to the innate immune response, defense response, and antigen presentation categories.

To further validate the role of cancer cell-derived extracellular RNASET2 in suppressing tumor growth in vivo, an engineered human RNASET2 protein carrying a KDEL endoplasmic reticulum retention motif, in order to prevent its secretion, was expressed in HEY3MET2 cells before challenging them in nude mice. Strikingly, unlike cells overexpressing wild-type RNASET2, which were significantly inhibited in their tumorigenic potential, cells overexpressing the KDEL-modified RNASET2 protein gave rise to large tumors, which reached the same size as RNASET2-negative cells derived from empty-vector transfection. This result strongly suggested that RNASET2-mediated tumor suppression in vivo is largely dependent on extracellular RNASET2 [103].

Xenograft-based cancer models rely on immune-deficient animals; therefore, they are not suitable to define the role of a tumor suppressor gene in a fully immunocompetent host. To overcome this limitation, a syngeneic mouse model was developed by De Vito et al. by overexpressing the murine *Rnaset2* gene in C51 mouse colorectal cancer cells, followed by injection of the engineered cells into BALB/c mice. Noteworthy, the tumor growth rate was significantly decreased in mice injected with Rnaset2-expressing cells when compared to those receiving the control empty vector-transfected cells. Moreover, a significant increase in tumor-free survival was observed in mice injected with *Rnaset2*-expressing cells [87].

Interestingly, immunohistochemical analysis of tumor samples showed marked recruitment of CD86^+^ macrophages in Rnaset2-expressing tumors.

In an independent experiment, C51 control or Rnaset2-overexpressing cells were inoculated in BALB/c mice to investigate their ability to withstand a rechallenge with parental C51 cells. As expected, several mice injected with Rnaset2-overexpressing cells could completely reject tumor cells. Strikingly, when these mice were rechallenged with parental C51 cells, unlike the control mice, 75% of them had become able to reject these cells, suggesting the occurrence of an immune memory response in these animals, likely induced by the previous exposure to Rnaset2-overexpressing cells (Figure 2D) [87].

A subsequent immunohistochemical analysis of the tumors from these mice showed a significant Rnaset2-mediated rebalance of an intra-tumor M1/M2 host macrophage population, since an increase in M1 infiltrating macrophage was observed, coupled with a marked decrease in M2 macrophages. Moreover, the number of myeloid-derived suppressor cells (MDSCs) was significantly reduced in tumors from mice injected with *Rnaset2*-expressing cells, coupled with a decrease in CD31^+^ cells, suggesting an additive effect of murine Rnaset2 in inhibiting angiogenesis. Of note, a five-fold increase in CD8^+^ T cells was also found in these mice, suggesting a potential Rnaset2-mediated oncosuppressive role carried out by these cells as effector cytotoxic immune cells (Figure 2D) [87].

Taken together, these data strongly point at human RNASET2 as a powerful modulator of the innate immune system endowed with the ability to mount an effective antitumoral response in vivo.

## 8. Discussion

T2 RNases represent a very ancient class of ribonucleases endowed with a host defense role. Two decades of intensive research pointed at T2 RNases as very promising molecules in the context of innovative cancer therapy. Indeed, in addition to the long-established role of these enzymes as wide-range tumor suppressors acting in a cell-autonomous manner, recent data have clearly unveiled a second, possibly more powerful, anticancer activity carried out by these proteins through their extracellular, non-cell-autonomous roles in the context of the TME. Several studies have consistently reported that T2 RNases play a critical role in suppressing angiogenesis in both in vitro and in vivo experimental models.

Even more appealing is the recent discovery of a widespread role of T2 RNases in modulating both innate and adaptive immunity to trigger an oncosuppressive response that, apparently, calls into play several cellular effectors from this defense system, including monocytes, macrophages, dendritic cells, granulocytes, and T lymphocytes.

The biological relevance of these T2 RNase-mediated oncosuppressive responses in the TME is further highlighted by their extremely high evolutionary conservation, ranging from invertebrate to vertebrate model systems.

The translational potential of human RNASET2 for innovative cancer therapy is also supported by the frequent finding of changes in the expression levels of this protein in human cancer specimens corresponding to different stages of grades. In several instances, RNASET2 levels were found to be decreased in advanced tumors, such as ovarian cancer, gastric cancer, and acute lymphocytic leukemia, as expected for a classical tumor suppressor gene and/or an alarmin-like molecule [95,122]. On the other hand, in other cancer types, such as neuroendocrine lung neoplasms (Lu-NENs), RNASET2 expression was found to be upregulated in high-grade tumor samples [123]. Though counterintuitive, this finding is likely related to the fact that human RNASET2 is known to be expressed by both cancer cells and TME-derived immune cells. Thus, in some cancer types, the upregulation of RNASET2 in high-grade/stage samples may simply represent a persistent, stress-induced biological response of the TME based on increased expression of the alarmin-like T2 RNase by immune cells to stimulate an oncosuppressive immune response.

In keeping with this hypothesis, immunohistochemical analysis of high-grade, RNASET2-expressing Lu-NENs revealed a preferential expression of this protein in tumor-infiltrating inflammatory cells, morphologically consistent with tissue macrophages. Moreover, high-grade Lu-NENs were characterized by a significant increase in apoptosis, coupled with a lower microvessel density, two biological responses that have long been linked to increased RNASET2 expression [123].

In the last decades, TME-based approaches for cancer therapy have gained increasing attention, due to the growing awareness of the key role played by stromal components within a tumor mass in cancer growth regulation. Two key processes in cancer development and progression that have been thoroughly targeted are represented by angiogenesis and immune surveillance escape. However, even after decades of intensive research, targeting cancer blood vessels with angiogenesis inhibitors alone usually resulted in a rather low benefit for most cancer types.

On the other hand, cancer immunotherapy is currently seen as a frontline research field for cancer therapy, and immune checkpoint inhibitors are now widely used for the treatment of several cancer types [124]. Nonetheless, several challenges still need to be addressed for this therapeutic approach as well [42].

Within this frame, we reckon that T2 RNases (and human RNASET2 in particular), due to their established “multitasking” oncosuppressor activity carried out at both cell-autonomous and non-cell-autonomous levels, might represent a very promising and innovative anti-cancer tool. Indeed, the bulk of experimental evidence in preclinical models described above has consistently shown a marked effect of RNASET2 in in vivo tumor suppression.

It is tempting to speculate that the high efficacy of T2 RNases in repressing cancer growth may be ascribed to their peculiar and redundant roles in both cancer cells and cells belonging to the TME. Thus, in those cases where the mutational burden of tumor cells makes them able to bypass the cell-autonomous oncosuppressive role of the T2 RNase protein they produce, the same protein secreted in the TME (by either cancer cells themselves or by surrounding immune cells) may orchestrate a powerful and independent anti-cancer response driven by both the suppression of angiogenesis and the activation of a powerful innate and possibly adaptive anticancer immune response.

## 9. Conclusions

The identification of molecules as possible biomarkers to trace pathology insurgence, dynamics, and fate, still represents a challenge and a relevant clinical unmet need. By definition, biomarkers should be specific to a given pathology, stable, robust, and easily detected. Based on this latter concept, the possibility to detect molecules of interest in easy-to-access biofluids, such as saliva, urine, and blood, further represents a challenge.

Here, while being conscious that the full impact of RNASET2 in the clinical setting still requires further elucidation, we highlighted several biological roles of RNASET2, which acts in cell-autonomous and non-cell-autonomous manners, by pointing out its influence on the pathogenesis of various disorders, including neurodegeneration, cancer, and infections, suggesting its promising implications for future possible clinical applications. Even if the complete impact on human health and disease remains partially undisclosed, in this review, we highlighted several biological roles of RNASET2 in “distant” pathologies that share inflammation as a hallmark.

Moving to the tumor context and considering the well-established role of RNASET2 in tumors and the tumor microenvironment (the latter including both the stromal and the immune compartments in tumors), together with its impact on the cancer therapy response, we suggest that RNASET2 can be envisaged as a marker for early tumor response, based on its alarmin-like actions, as well as a marker for induced anti-tumor immune response, following therapies potentially re-activating the immune system against tumor cells. Also, given the differential expression of RNASET2 during the development and progression of various diseases, this molecule might be considered as a potential diagnostic biomarker. Measuring RNASET2 levels in patient samples, including blood (within a liquid biopsy approach) or tissue, might aid in disease detection, prognosis, and monitoring treatment responses. Finally, RNASET2 has also demonstrated antiviral, anti-bacterial, and anti-parasite activity, inhibiting the replication of certain viruses, thus suggesting a potential use for the development of antiviral/antibacterial drugs.

Additional research and clinical trials are necessary to better understand the regulatory mechanisms of RNase T2 in chronic diseases. These studies will pave the way for the development of innovative diagnostic and therapeutic approaches centered on RNASET2.

## Data Availability

Not applicable.
